# Analyzing the relationship between socioeconomic deprivation and outpatient Medicare Part D fluoroquinolone claim rates in Texas

**DOI:** 10.1017/ash.2024.38

**Published:** 2024-04-01

**Authors:** Mayar Al Mohajer, Edgar Samarasundera, Judite Gonçalves, Alicia Heath

**Affiliations:** 1 Department of Medicine, Baylor College of Medicine, Houston, TX, USA; 2 Department of Primary Care and Public Health, School of Public Health, Imperial College London, London, UK; 3 NOVA National School of Public Health, Public Health Research Centre, Comprehensive Health Research Center, CHRC, NOVA University Lisbon, Lisbon, Portugal; 4 Department of Epidemiology and Biostatistics, School of Public Health, Imperial College London, London, UK

## Abstract

**Introduction:**

Only a few studies have assessed the relationship between deprivation and excessive antibiotic use. In Texas, antimicrobial prescription rates are particularly high compared with the rest of the US. This study analyzed the association between local area socioeconomic deprivation and providers’ fluoroquinolone claim rates among beneficiaries 65 years and older in Texas.

**Methods:**

This ecological study utilized provider- and area-level data from Medicare Part D Prescribers and the Social Deprivation Index (SDI) repositories. Negative binomial regression models were employed to evaluate the relationship between provider- and area-level characteristics (prescriber’s gender, specialty, rural-urban community area, beneficiaries’ demographics, area-level population, and SDI) and fluoroquinolone claim rates per 1,000 beneficiaries.

**Results:**

A total of 11,996 providers were included. SDI (IRR 0.98, 95% CI 0.97–0.99) and male providers (IRR 0.96, 95% CI 0.94–0.99) were inversely associated with claim rates. In contrast, several factors were associated with higher claim rates, including non-metropolitan areas (1.04, 95% CI 1.00–1.09), and practices with a high proportion of male (IRR 1.12, 95% CI 1.10–1.14), Black (IRR 1.05, 95% CI 1.03–1.07), or Medicaid beneficiaries (IRR 1.15, 95% CI 1.12–1.17). Effect modification was observed between SDI and rurality, with higher SDI in non-metropolitan areas associated with higher claim rates, whereas SDI in metropolitan areas was inversely related to claim rates.

**Conclusion:**

Lower fluoroquinolone claim rates were observed among Texas Medicare providers in metropolitan areas with higher SDI. Conversely, higher rates were observed in rural areas with higher SDI. More studies are needed to understand the underlying causes of this variation and develop effective stewardship interventions.

## Introduction

Excessive antibiotic prescription is associated with allergic drug reactions, disruption of gut microbiota, antimicrobial resistance, *Clostridioides difficile (C. difficile)* infection, and increased mortality.^1,2^ In the United States (US), patients aged ≥65 years have the highest prescribing rate of any age group.^3^ They are 50% more likely to receive antimicrobials than younger adults^4^ and are at an increased risk for antibiotic-associated adverse events.^1^

Fluoroquinolones are commonly prescribed in the outpatient setting due to their excellent bioavailability, broad spectrum of activity, and easy dosing;^5^ however, they are associated with several side effects, particularly in older patients.^6^ In addition to antimicrobial resistance and high rates of *C. difficile*, their excess use in this population has been linked to an increased risk of tendinopathy, aortic dissection, and cardiac arrhythmia.^7^ This has prompted the Food and Drug Administration to impose box warnings to this antibiotic class and recommend against their use for uncomplicated infections.^8^ Despite these recommendations, it is estimated that 5.1% of ambulatory fluoroquinolone prescriptions are given when no antimicrobials are needed, and 19.1% are ordered when an alternate first-line antimicrobial could be prescribed.^9^

Prescribing patterns of antimicrobials in older patients can vary by region and provider specialty. One study^10^ analyzed the Centers for Medicare and Medicaid Services (CMS) Part D Prescriber database,^11^ which comprises administrative claims of beneficiaries (patients) aged ≥65 years enrolled in the Part D Medicare program (70% of the total Medicare population). Most (48.1%) high-volume prescribers (top 10%) were in the US South, including Texas.

This variation in prescribing antimicrobials is related to healthcare providers’ prescription patterns^10^ and patient demand for antibiotics.^12^ These two interrelated factors are influenced by external ones, such as knowledge about the risks and benefits of antibiotics, access to care, and socioeconomic factors.^12–14^

In the US, studies have demonstrated the complex relationship between socioeconomic factors and antibiotic use. Low education,^13^ poverty,^13^ and physician density^14^ may all play a role in antibiotic overprescription in the South of the US. Conversely, low income may lead to lower prescription rates due to lack of access^14,15^ with subsequent use of non-prescribed antimicrobials.^16^ Existing studies varied in geographical coverage, patient population, care setting, methodology, outcome definition, and variables included, limiting comparability and external validity.

Notably, there remains a gap in the literature regarding fluoroquinolone use among older individuals, particularly in the US South, where misuse is high.^10^ This is particularly important to address, given the high rates of fluoroquinolone side effects and the frequent inappropriate use of this class of antimicrobials. This study aimed to analyze the association of area-level deprivation and its components with provider Medicare claims for fluoroquinolones in the outpatient setting in Texas.

## Methods

### Data sources and access

This ecological study utilized two repositories of data collected at different geographic levels. These included the Medicare Part D Prescribers data (provider level) and the SDI repository (ZIP Code Tabulation Area [ZCTA] level).

The CMS dataset for fiscal year 2021^11^ comprises antimicrobial, opioid, and antipsychotic claims of beneficiaries enrolled in the Part D Medicare program. Medicare is the public insurance program in the US that covers individuals 65 years and older or with disability. Part D is the subprogram covering prescribed drug utilization. This repository contains information on providers, drug claims, and their beneficiaries. Provider variables include National Provider Identifiers, full names, gender, population density, Rural-Urban Commuting Area (RUCA) codes, and specialties. Drug claims data encompass the total number of claims by type (antibiotics, antipsychotics, and opioids). Beneficiary information comprises the total number of beneficiaries, average age, counts of demographics (age, gender, race, and ethnicities), dual insurance status (Medicare and Medicaid vs Medicare only), and risk score. The risk scores estimate patient spending and are calculated based on demographics, Medicaid eligibility, disability qualification, living in a nursing home, and previous diagnoses.^17^ Due to confidentiality concerns, CMS suppresses claim values for providers with fewer than eleven beneficiaries or fluoroquinolone claims.

The SDI repository^18^ includes aggregate-level (at the ZCTA level) socioeconomic measures of poverty, education, single-parenthood, rental housing, overcrowding, car ownership, and unemployment collected between 2015 and 2019. Each measure is converted from a percentage to a normalized score to calculate the final index.

### Socioeconomic context

This study’s main proxy for socioeconomic context was the SDI composite measure (at the ZCTA level) and its seven social determinants of health (SDOH) components (see above). These variables, including the composite SDI index, were provided on a scale from 1 to 100 (percentiles), where higher values indicate higher deprivation.

### Outcome

The primary outcome was the provider rate of Medicare Part D fluoroquinolone claims per 1,000 beneficiaries.

### Covariates

To account for possible confounding between SDI (or its components) and the rate of fluoroquinolone claims per beneficiary, this study adjusted for the following variables at the provider level: prescriber gender, specialty, RUCA, beneficiary average age, beneficiary average risk score, and percentages of beneficiaries who are female, Black, Hispanic, or with dual public insurance (Medicare and Medicaid). The prescriber specialty was grouped into six categories (medical, surgical, urology, pulmonary, infectious diseases, and others), while the RUCA was classified as a metropolitan or non-metropolitan area. In addition, the patient population size and the percentage of Black, Hispanic, with high needs, and foreign-born individuals were included at the ZCTA level to adjust for area-level differences.

### Statistical analysis

The Chi-squared test was used to compare characteristics between included and excluded providers. The Kruskal-Wallis test was utilized to compare the median rates of fluoroquinolone claims per 1,000 beneficiaries across categorical variables, including, for simplicity, SDI quintiles. To identify geographic patterns and autocorrelation within and between SDI and fluoroquinolone claims, spatial dependence of these two variables was assessed by Local Indicators of Spatial Association (LISA, both univariate and bivariate) cluster mapping along with the global and local Moran’s I analyses.

Multiple Imputation by Chained Equations was performed to fill in the missing data for patient and provider demographics, RUCA, and socioeconomic factors (with >10% missing), while imputation with median was applied for SDI and fluoroquinolone claim rates before spatial analyses. Sensitivity analyses were performed, excluding variables with large portions of missing values (>10%).

Negative binomial regression models for rates were fitted to estimate incidence rate ratios (IRRs) and 95% confidence intervals (CIs) for the relationship between SDI (or its components), as well as covariates, and the rate of fluoroquinolone claims per 1,000 beneficiaries.

In the first model (M1), fixed factors (patient and provider demographics, specialty, risk score, dual insurance status, metropolitan area, and SDI) were added a priori to the null model (M0). Continuous variables were normalized before being entered. The second model (M2) incorporated an interaction term between SDI and metropolitan area.

The third model (M3) comprised all non-collinear normalized SDOH scores (rather than the composite SDI) and the normalized scores for Black and Hispanic individuals, those with high needs, and those foreign-born. The final model (M4) additionally included an interaction term between the metropolitan area and unemployment due to the known relationship between these variables.^19^ Multilevel modeling was tested for clustering across ZCTAs (random intercept) and SDI nested within ZCTAs (random slope).

Model fit was assessed by plotting the scaled residuals via the Residual Diagnostics for Hierarchical Models package. *P*-values <0.05 were considered statistically significant. The analysis was conducted in R version 4.2.2 (Vienna, Austria).

## Results

### Study characteristics

A total of 65,299 providers in Texas within 1,280 ZCTAs were identified. After excluding providers with fewer than 11 beneficiaries or fluoroquinolone claims, 11,996 providers within 968 ZCTAs were included for analysis. The provider characteristics are presented in Table 1.

**Table 1.** Characteristics of included and excluded providers

Provider characteristics	Included providers (n = 11,996)	Excluded providers^a^ (n = 72455)	*P*-value
	n (%)	n (%)	
Gender			
Female	4834 (40.3%)	34363 (47.4%)	<0.001
Male	7162 (59.7%)	38092 (52.6%)	
Specialty			
Infectious diseases	127 (1.1%)	237 (0.3%)	<0.001
Medical	8663 (72.2%)	33970 (46.9%)	
Pulmonary	186 (1.6%)	537 (0.7%)	
Surgical	230 (1.9%)	7086 (9.8%)	
Urology	538 (4.5%)	173 (0.2%)	
Others	2252 (18.8%)	30452 (42.0%)	
Metropolitan area- Yes	10400 (86.7%)	67278 (92.9%)	<0.001

a Providers were excluded if they had fewer than 11 patients or claims, given the Centers for Medicare and Medicaid Services’ suppression of data, and thus, relevant information was not available for analysis.

The number of prescribing providers did not vary significantly across each deprivation quintile (Table 2). The median patient age in the prescribing practices was 72.7 years; most beneficiaries were female (58.9%) and White (69.8%). Less than a quarter (22.7%) had dual insurance.

**Table 2.** Characteristics of patients, deprivation quintiles, and fluoroquinolone claim rates

Characteristics	
Patients	Median (IQR)
Total beneficiaries per provider	332 (198–526)
Average beneficiary age (years)	72.7 (71.0–74.2)
% Female beneficiaries per provider	58.9 (53.4–64.8)
% White beneficiaries per provider	69.8 (44.9–82.6)
% Black beneficiaries per provider	10.4 (5.5–18.9)
% Hispanic beneficiaries per provider	15.0 (7.9–38.6)
% Other ethnicities per provider	6.4 (0–11.6)
% Beneficiaries with dual insurance	22.7 (12.2–37.7)
Risk score	1.4 (1.1–1.9)
SDI^a^	n (%)
Providers in quintile 1 -SDI [85,100] (most deprived)	2337 (19.5)
Providers in quintile 2 -SDI [71, 85)	2374 (19.8)
Providers in quintile 3 -SDI [48, 71)	2417 (20.1)
Providers in quintile 4 -SDI [29, 48)	2367 (19.7)
Providers in quintile 5 -SDI [1, 29) (least deprived)	2501 (20.8)
Claim Rates	Median (IQR)
Ciprofloxacin claims per provider	16 (0–28)
Levofloxacin claims per provider	0 (0–16)
Moxifloxacin claims per provider	0 (0–0)
Ofloxacin claims per provider	0 (0–0)
Total fluoroquinolone claims per provider^b^	24 (15–47)
Rate of claims/ 1000 beneficiaries per provider	88.8 (53.5–154.6)

Note. IQR, Interquartile Range; SDI, Social Deprivation Index.

a Providers were grouped into SDI quintiles.

b Fluoroquinolone claims included ciprofloxacin, levofloxacin, moxifloxacin, ofloxacin.

**Table 3.** Fluoroquinolone claim rates stratified by provider characteristics and SDI

Variable	Median fluoroquinolone claims per 1,000 beneficiaries (IQR)	*P*-value
Providers		
Gender:		<0.001
Female	82.4 (51.9–138.0)	
Male	94.5 (54.6–167.0)	
Specialty:		<0.001
Infectious diseases	211.0 (143.0–327.0)	
Medicine	78.8 (49.4–129.0)	
Pulmonary	74.9 (49.5–119)	
Surgery	129.0 (85.0–244.0)	
Urology	178.0 (114.0–275.0)	
Others	130.0 (64.4–268.0)	
Metropolitan area:		0.979
Yes	88.5 (53.1–156.0)	
No	92.0 (55.1–144.0)	
SDI^a^		<0.001
Quintile 1 –SDI [85,100] (most deprived)	87.6 (51.9-157.0)	
Quintile 2 -SDI [71, 85)	93.5 (56.6–161.0)	
Quintile 3 -SDI [48, 71)	86.3 (50.9–148.0)	
Quintile 4 -SDI [29, 48)	93.5 (56.6–161.0)	
Quintile 5 -SDI [1, 29) (least deprived)	83.3 (51.5–140.0)	

Note. IQR, Interquartile Range; SDI, Social Deprivation Index.

a Providers were grouped into SDI quintiles.

**Figure 1.**
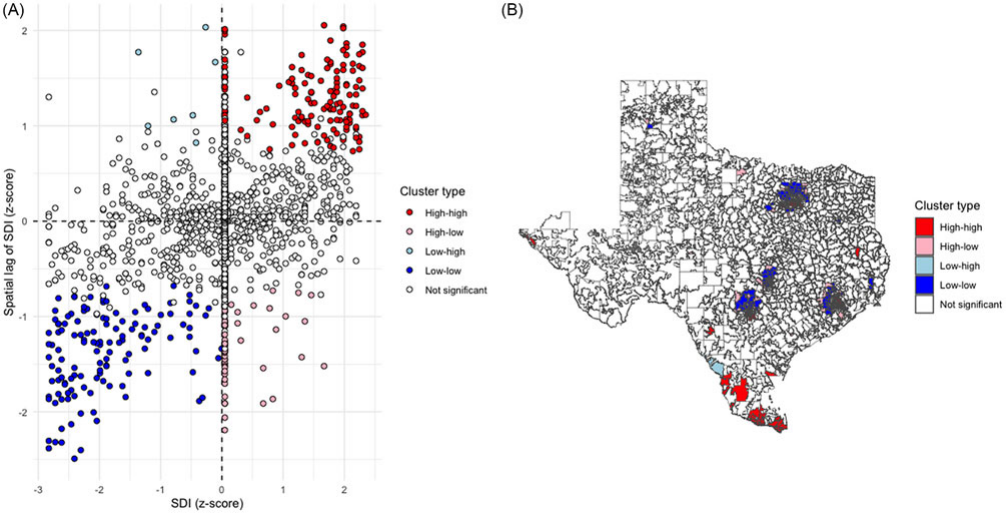
Spatial distribution and empirical Bayes ZCTA clusters of SDI in Texas. A- LISA Quadrant Scatter Plot B- LISA Cluster Map. The distribution included 161 high-high, 54 high-low, 7 low-high, 129 low-low, and 1577 non-significant clusters. High-high clusters were largely located in larger cities (Houston, Dallas, San Antonio, and Austin). Low-low clusters were mainly seen in Southern Texas (Valley area). SDI, Social Deprivation Index; ZCTA, Zip Code Tabulation Areas; LISA, Local Indicators of Spatial Association.

There was significant spatial clustering of SDI in Texas (Moran’s I global statistic 0.36, *P* < 0.001). A total of 161 ZCTAs with high SDI adjacent to other communities with high SDIs (high-high) were identified using local Moran’s I, compared to 129 low-low SDI ZCTAs (Figure 1).

### Fluoroquinolone prescription rates

The median number of fluoroquinolone claims per provider was 24 (interquartile range [IQR] 15–47), while the median rate of claims per 1,000 beneficiaries was 88.8 (IQR 53.5–154.6) (Table 2). Ciprofloxacin was the most commonly prescribed antimicrobial.

**Figure 2.**
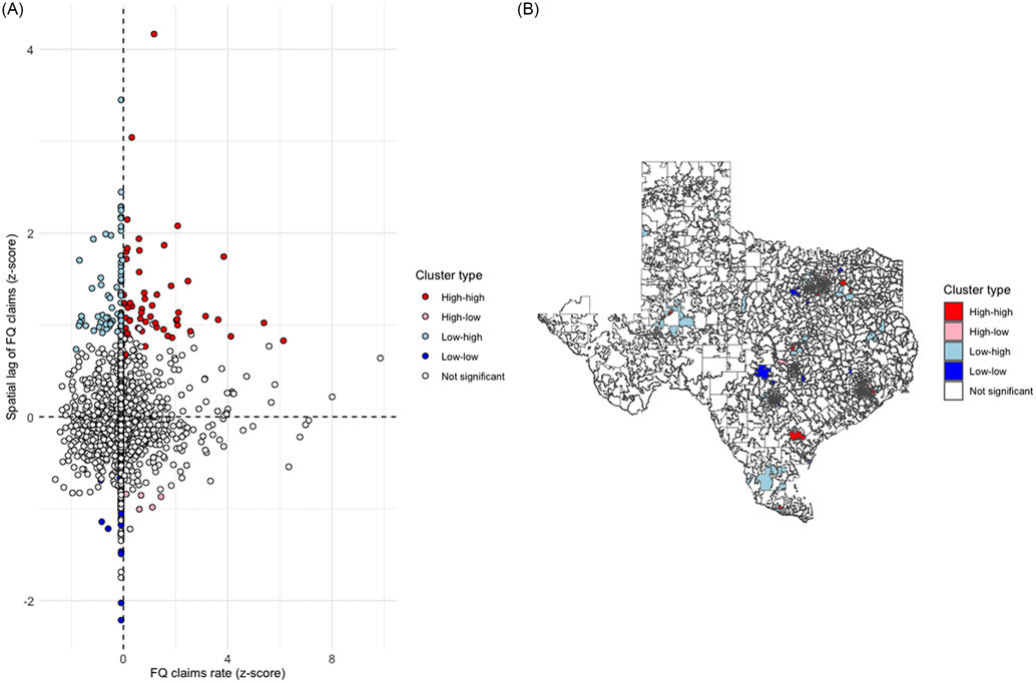
Spatial distribution and empirical Bayes ZCTA clusters of fluoroquinolone claim rates in Texas. A- LISA Quadrant Scatter Plot B- LISA Cluster Map. The distribution included 55 high-high, 5 high-low, 73 low-high, 20 low-low, and 1775 non-significant clusters. LISA, Local Indicators of Spatial Association; ZCTA, ZIP Code Tabulation Areas; FQ, Fluoroquinolones.

The global Moran’s *I* test identified significant spatial clustering of ZCTA fluoroquinolone claim rates (statistic 0.04, *P* = 0.002). Figure 2 shows the spatial distribution of claim rates, with 55 high-high and 20 low-low ZCTAs.

Fluoroquinolone claim rates varied by provider characteristics and SDI (Table 3). There was little difference in prescribing rates between providers in metropolitan (88.5 per 1,000 beneficiaries) and non-metropolitan areas (92.0 per 1,000 beneficiaries, *P* = 0.979). Infectious disease physicians had the highest claim rates (211.0 per 1,000 beneficiaries). The prescription rate varied by deprivation quintile (*P* < 0.001), and the least deprived quintile had the lowest claim rate.

### Factors associated with fluoroquinolone claim rates

**Figure 3.**
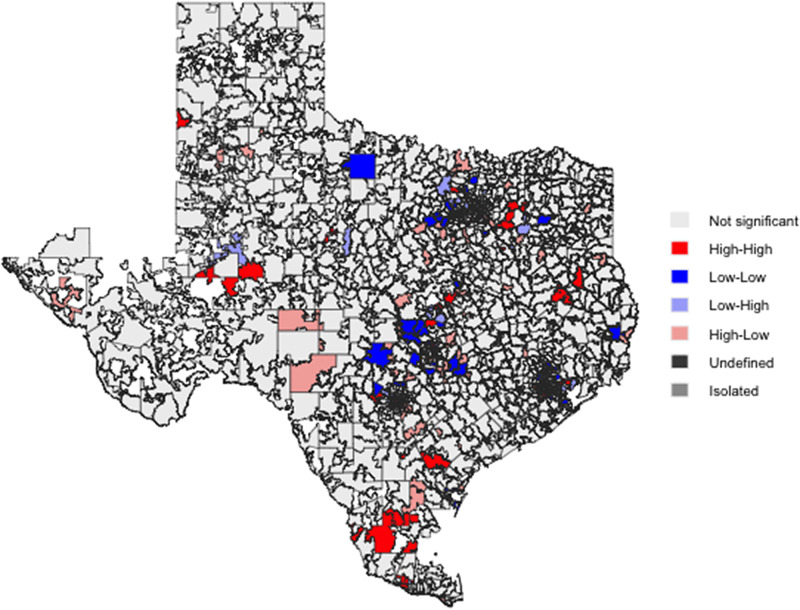
Bivariate LISA Map of SDI and fluoroquinolone claims per 1,000 Beneficiaries Across Texas ZCTAs. The distribution included 85 high-high, 38 low-low, 45 low-high, 93 high-low, and 1667 non-significant clusters. Global Moran’s test showed no spatial dependence between SDI and rates of fluoroquinolone claims in Texas (I = 0.01, *P* = 0.618). LISA, Local Indicators of Spatial Association; SDI, Social Deprivation Index; ZCTA, ZIP Code Tabulation Areas.

There was no spatial dependence between SDI and rates of fluoroquinolone claims in Texas (Global Moran’s I = 0.01, *P* = 0.618). Bivariant LISA maps (Figure 3) showed 85 high-high and 38 low-low spatial clusters.

Results for the negative binomial analysis are presented in Table 4. In the first model (M1), medical specialties, pulmonologists, and urologists had lower fluoroquinolone claim rates per 1,000 beneficiaries than surgical specialties. Lower SDI, female providers, non-metropolitan areas, ZCTAs with smaller populations, older beneficiaries, and practices with a high proportion of male, Black, or dual insurance beneficiaries were associated with higher claim rates. The second model (M2) showed that the negative relationship between SDI and claim rates observed in M1 was driven by urban areas, whereas in rural areas, higher SDI was associated with higher fluoroquinolone claims per beneficiary (IRR 1.13, 95% CI 1.07–1.21).

**Table 4.** Incidence rate ratios and 95% confidence intervals for rates of fluoroquinolone claims in relation to socioeconomic deprivation and its components and provider characteristics: results from negative binomial regression models

	M0			M1			M2			M3			M4		
Predictors	IRR	95% CI	*P*	IRR	95% CI	*P*	IRR	95% CI	*P*	IRR	95% CI	*P*	IRR	95% CI	*P*
(Intercept)	0.13	0.12–0.13	**<0.001**	0.21	0.19–0.23	**<0.001**	0.22	0.20–0.26	**<0.001**	0.24	0.21–0.27	**<0.001**	0.23	0.20–0.27	**<0.001**
Metro [Rural, ref Metro]				1.04	1.00–1.09	**0.034**	1.01	0.96–1.05	0.739	1.08	1.03–1.13	**0.002**	1.05	1.01–1.11	**0.027**
Gender [Male, ref Female]				0.96	0.94–0.99	**0.015**	0.97	0.94–0.99	**0.020**	0.97	0.94–1.00	**0.025**	0.97	0.94–1.00	**0.029**
Specialty [ID, ref Surgery]				1.07	0.92–1.26	0.373	1.07	0.92–1.26	0.377	1.08	0.93–1.27	0.322	1.08	0.92–1.27	0.323
Specialty [Medical]				0.48	0.44–0.53	**<0.001**	0.49	0.44–0.53	**<0.001**	0.49	0.45–0.54	**<0.001**	0.49	0.45–0.54	**<0.001**
Specialty [Others]				0.98	0.89–1.09	0.733	0.98	0.89–1.08	0.715	0.99	0.89–1.09	0.776	0.98	0.89–1.09	0.750
Specialty [Pulmonary]				0.46	0.40–0.53	**<0.001**	0.46	0.40–0.53	**<0.001**	0.47	0.41–0.54	**<0.001**	0.47	0.41–0.54	**<0.001**
Specialty [Urology]				0.83	0.73–0.94	**0.003**	0.83	0.73–0.93	**0.003**	0.83	0.73–0.93	**0.003**	0.82	0.73–0.93	**0.002**
Age (years)				1.09	1.08–1.11	**<0.001**	1.09	1.08–1.11	**<0.001**	1.09	1.07–1.11	**<0.001**	1.09	1.07–1.11	**<0.001**
Risk score				1.01	1.00–1.03	0.089	1.01	1.00–1.03	0.093	1.01	0.99–1.03	0.252	1.01	0.99–1.03	0.249
% Male patients				1.12	1.10–1.14	**<0.001**	1.12	1.10–1.14	**<0.001**	1.12	1.10–1.14	**<0.001**	1.12	1.10–1.14	**<0.001**
% Black patients				1.05	1.03–1.07	**<0.001**	1.05	1.03–1.06	**<0.001**	1.04	1.02–1.06	**<0.001**	1.04	1.02–1.06	**<0.001**
% Hispanic patients				0.99	0.97–1.01	0.345	0.99	0.97–1.01	0.293	1.01	0.99–1.04	0.207	1.01	0.99–1.04	0.290
% dual beneficiary patients				1.15	1.12–1.17	**<0.001**	1.14	1.12–1.17	**<0.001**	1.15	1.12–1.17	**<0.001**	1.15	1.12–1.17	**<0.001**
Population size				0.95	0.94–0.97	**<0.001**	0.95	0.94–0.97	**<0.001**	0.96	0.95–0.98	**<0.001**	0.96	0.95–0.98	**<0.001**
SDI^a^				0.98	0.97–0.99	**0.005**	0.97	0.96–0.99	**<0.001**						
Metro [Rural] × SDI							1.13	1.07–1.21	**<0.001**						
% Below FPL										1.03	0.99–1.06	0.135	1.03	1.00–1.07	0.056
% Single house										0.99	0.96–1.02	0.397	0.99	0.96–1.02	0.498
% Dropout										1.02	0.99–1.05	0.207	1.02	0.99–1.06	0.138
% No car										0.97	0.94–0.99	**0.014**	0.97	0.94–0.99	**0.007**
% Renter occupied										1.04	1.01–1.07	**0.002**	1.04	1.01–1.06	**0.004**
% Crowded units										0.97	0.94–1.00	**0.028**	0.97	0.94–0.99	**0.017**
% Unemployed										0.97	0.95–0.99	**0.001**	0.96	0.94–0.98	**<0.001**
% High needs										0.99	0.98–1.01	0.438	0.99	0.98–1.01	0.391
% Black										1.03	1.01–1.04	**0.005**	1.02	1.01–1.04	**0.008**
% Hispanic										0.97	0.95–1.00	**0.033**	0.98	0.95–1.00	**0.038**
% Foreign born										1.01	0.99–1.02	0.495	1.01	0.99–1.03	0.459
Metro [Rural] × % Unemployed													1.06	1.02–1.10	**0.004**
Observations	11,996			11,996			11,996			11,996			11,996		
R^2^	0.000			0.321			0.323			0.326			0.327		

Note. IRR: Incidence Rate Ratio; CI: Confidence Interval; Metro: Metropolitan area; ID: Infectious Diseases; SDI: Social Deprivation Index; FPL: % Federal Poverty Level.

The first model (M1) contained the SDI, the population size, and provider covariates. The second model (M2) added an interaction term between SDI and the metropolitan area. In the third model (M3) all non-collinear normalized SDOH scores, and scores for %Black, %Hispanic, %High needs, and %Foreign born were included rather than the composite SDI. Finally, the fourth model (M4) incorporated an interaction between unemployment and the metropolitan area.

Bold signifies statistical significance (<0.05).

a SDI is measured as a continuous variable (0–100) and then normalized. Thus, estimated IRRs are per one standard deviation increment in SDI.

In the third model (M3), which replaces the overall SDI index by its components, the proportions of Black patients and households living in rented housing in the ZCTA were positively associated with the rate of fluoroquinolone claims. Conversely, the proportions of Hispanics, unemployed, households with no vehicle, and crowded households were inversely associated with claim rates. In non-metropolitan areas, the proportion of unemployed in the ZCTA was positively associated with fluoroquinolone claim rates (M4), in line with the findings of M2.

Sensitivity analyses (Supplementary Table 1) showed similar results when the two variables with >10% missing were excluded (proportions of Black and Hispanic patients in the practice). The simulated dispersion test for the models (M0-M4) revealed *P* values >0.05, signifying the absence of overdispersion or underdispersion. The multilevel modeling had comparable results (Supplementary Table 2) with an intraclass correlation coefficient (ICC) of only 1%, indicating a minimal role of clustering in explaining variation in antibiotic claims rates.

## Discussion

This ecological study of FY2021 Medicare Part D fluoroquinolone claims in Texas revealed that the overall claim rates per 1,000 beneficiaries were lower in areas with higher SDI after adjusting for confounders. This relationship varied between metropolitan and non-metropolitan areas and by SDOH factors. In urban areas, higher SDI or unemployment was associated with lower claim rates. Conversely, the associations of SDI or unemployment with claim rates were positive in rural areas.

The spatial analyses showed that the distribution of high and low SDI and rates of fluoroquinolone claims were more geographically clustered than expected by random chance alone. The clusters of communities with low SDI (low-low) were in large cities, while those with high SDI (high-high) were in Southern Texas. However, the communities with high and low rates of fluoroquinolone claims were largely different from those with high and low SDI, leading to no bivariate spatial association. This suggests that the relationship between SDI and claim rates was not affected by the local segregation in the ZCTA distribution. Moreover, the low ICC result from the multilevel model (1%) confirms the low variability of claim rates within ZCTAs when adjusted for SDI.

Notably, this study highlighted the complex relationship between components of the deprivation index and fluoroquinolone claim rates. Some SDOH factors had a positive association with claims rates (prevalence of Blacks and rental housing). In contrast, others (prevalence of Hispanic ethnicity, unemployment, no car ownership, and crowded housing) were associated with lower claim rates.

This is the second study that analyzed the relationship between SDI and antimicrobial use in the US. Kissler et al.^15^ evaluated outpatient visit rates for respiratory tract infections among patients younger than 65 years in Massachusetts. They found that visit rates were lower in deprived areas, indicating possible underutilization of health services. Our study showed lower antibiotic claim rates in more deprived areas in a different population (Medicare beneficiaries residing in Texas). However, the lower claim rates among deprived subpopulations were principally seen in urban areas in Texas; this factor (metropolitan area) was not assessed in the former study. We could not determine the rate of outpatient visits, limiting our ability to assess the underutilization of health services. Notably, underutilizing services is likely a more significant problem in Texas as it is ranked 47^th^ in the country for physician access, compared with Massachusetts (ranked first).^20^

Klein et al.^14^ evaluated the impact of rurality and unemployment on outpatient antibiotic prescription rates in the US. Similar to our study, unemployment was associated with lower prescription rates. On the other hand, unlike our findings, they showed that rurality had a negative association with the prescription rate. This could be partly due to confounding as they did not adjust for other SDOH (eg, no car, crowding) in their analysis.

This study has some limitations. The main one is that antimicrobial prescribing rates in our data are blind to antimicrobial appropriateness, as the claim data did not include information on indications (eg, urinary tract infection, sinusitis). We can speculate that very high rates include inappropriate prescriptions and that very low rates may signify suboptimal use. However, we do not know the extent of over or underprescription. This would require detailed patient-level diagnosis data. A second limitation is that while the claims data pertain to FY2021, the SDI is based on 2015–2019 data. However, this is unlikely to be a major concern as the SDI remains relatively stable in the short term. Several variables in the CMS dataset have missing values and data for providers with less than 11 patients or claims were suppressed. We accounted for missingness using multiple imputations, and the sensitivity analysis excluding variables with large (>10%) portions of missing data provided concordant results. Still, our study is not representative of small providers in Texas (ie, those with fewer than 11 patients or claims). It is also unknown whether the relationship between SDI and antimicrobial prescription rates (particularly in rural vs urban areas) is similar in other locations outside Texas. Although our study adjusted for a comprehensive range of provider- and area-level indicators, it was not possible to account for other potentially important covariates (eg, provider and patient knowledge of antimicrobials, patient expectation of antimicrobial receipt, patient comorbidities, and access to care). Finally, given the study’s ecological nature, a temporal link between variables and the outcome cannot be established.

A key strength of this study is utilizing the SDI, a well-validated measure of deprivation, providing evidence of its usefulness for research exploring the role of local area deprivation for healthcare access. Importantly, we unpacked the SDI to look at the various SDOH components, revealing complex relationships, and explored key interactions with rurality.

Our study provides valuable information for public health officials in Texas. It highlighted variations in outpatient Medicare fluoroquinolone claim rates by geography, provider specialties, and SDI. This could enable allocating resources for targeted antimicrobial stewardship interventions to limit excessive use. In addition, the study identified several SDOH domains associated with variation in claim rates, either positively or negatively. These findings provide opportunities for public health professionals to explore gaps in the knowledge and attitudes of patients and providers related to antimicrobial use, particularly in rural regions, and investigate barriers to healthcare access in metropolitan areas. More studies are needed to explore how socioeconomic and other cultural factors impact patients’ and providers’ knowledge, attitudes, and practices toward antimicrobials and how access to care affects antimicrobial prescription rates.

In conclusion, this study showed lower fluoroquinolone claim rates among patients aged ≥65 years in urban ZCTAs in Texas with higher SDI and unemployment. However, in rural areas, higher claim rates were seen in ZCTAs with higher SDI and unemployment. Therefore, new stewardship interventions should be location-specific (rural vs urban) and target the drivers for fluoroquinolone over or underprescription in this population.

## Supporting information

Al Mohajer et al. supplementary material Al Mohajer et al. supplementary material

## Supplementary material

For supplementary material accompanying this paper visit http://doi.org/10.1017/ash.2024.38. click here to view supplementary material
