# Supplementary material for: Analyzing the relationship between socioeconomic deprivation and outpatient Medicare Part D fluoroquinolone claim rates in Texas
Source: Antimicrob Steward Healthc Epidemiol. 2024 Apr 1;4(1):e40. doi: 10.1017/ash.2024.38 (PMC10983052; doi:10.1017/ash.2024.38)
Supplement: Al Mohajer et al. supplementary material [file S2732494X2400038Xsup001.docx]

**Appendix**

**Appendix Table 1. Incidence rate ratios and 95% confidence intervals for rates of fluoroquinolone claims from negative binomial regression after excluding variables with > 10% missing**

|  | **Fluroquinolones claim rate per beneficiary** | | |
| --- | --- | --- | --- |
| *Predictors* | *Incidence Rate Ratio* | *95% CI* | *p* |
| (Intercept) | 0.23 | 0.20 – 0.26 | **<0.001** |
| Metro imp | 0.96 | 0.93 – 1.00 | 0.070 |
| Gender [Male, ref Female] | 0.96 | 0.93 – 0.99 | **0.010** |
| Specialty [ID, ref Surgery] | 0.45 | 0.40 – 0.52 | **<0.001** |
| Specialty [Medical] | 0.92 | 0.81 – 1.05 | 0.230 |
| Specialty [Others] | 0.44 | 0.37 – 0.51 | **<0.001** |
| Specialty [Pulmonary] | 0.94 | 0.80 – 1.10 | 0.434 |
| Specialty [Urology] | 0.78 | 0.67 – 0.90 | **0.001** |
| Age (years) | 1.08 | 1.06 – 1.09 | **<0.001** |
| Risk score | 1.02 | 1.01 – 1.04 | **0.004** |
| Prop. Female in practice | 0.90 | 0.88 – 0.91 | **<0.001** |
| Prop. with Medicaid in practice | 1.14 | 1.12 – 1.16 | **<0.001** |
| Population | 0.95 | 0.94 – 0.96 | **<0.001** |
| SDI | 0.98 | 0.96 – 0.99 | **0.001** |
| Observations | 11996 | | |
| R^2^ Nagelkerke | 0.316 | | |

CI: Confidence Interval; Metro: Metropolitan area; ID: Infectious Diseases; SDI: Social Deprivation Index

**Appendix Table 2. Multilevel modeling for rates of fluoroquinolone claims**

|  | **MLM1** | | | **MLM2** | | | **MLM3** | | | **MLM4** | | | **MLM5** | | |
| --- | --- | --- | --- | --- | --- | --- | --- | --- | --- | --- | --- | --- | --- | --- | --- |
| Predictors | IRR | 95% CI | IRR | IRR | 95% CI | P | IRR | 95% CI | P | IRR | 95% CI | P | IRR | 95% CI | P |
| (Intercept) | 0.22 | 0.19 – 0.25 | **<0.001** | 0.22 | 0.19 – 0.25 | **<0.001** | 0.22 | 0.19 – 0.25 | **<0.001** | 0.22 | 0.19 – 0.25 | **<0.001** | 0.22 | 0.19 – 0.25 | **<0.001** |
| Metro [Rural, ref Metro] | 1.06 | 1.01 – 1.12 | **0.030** | 1.06 | 1.01 – 1.12 | **0.031** | 1.03 | 0.98 – 1.09 | 0.242 | 1.08 | 1.02 – 1.15 | **0.006** | 1.06 | 1.00 – 1.13 | **0.041** |
| Gender [Male, ref Female] | 0.97 | 0.94 – 1.00 | **0.037** | 0.97 | 0.94 – 1.00 | **0.037** | 0.97 | 0.94 – 1.00 | **0.042** | 0.97 | 0.94 – 1.00 | **0.037** | 0.97 | 0.94 – 1.00 | **0.042** |
| Specialty [ID, ref Surgery] | 0.92 | 0.78 – 1.07 | 0.271 | 0.92 | 0.78 – 1.07 | 0.271 | 0.92 | 0.78 – 1.07 | 0.273 | 0.91 | 0.78 – 1.07 | 0.256 | 0.91 | 0.78 – 1.07 | 0.258 |
| Specialty [Medical] | 0.45 | 0.39 – 0.51 | **<0.001** | 0.45 | 0.39 – 0.51 | **<0.001** | 0.45 | 0.39 – 0.51 | **<0.001** | 0.45 | 0.39 – 0.51 | **<0.001** | 0.45 | 0.39 – 0.51 | **<0.001** |
| Specialty [Others] | 0.88 | 0.77 – 1.00 | **0.043** | 0.88 | 0.77 – 1.00 | **0.043** | 0.88 | 0.77 – 1.00 | **0.043** | 0.87 | 0.77 – 1.00 | **0.042** | 0.87 | 0.77 – 0.99 | **0.041** |
| Specialty [Pulmonary] | 0.42 | 0.35 – 0.49 | **<0.001** | 0.42 | 0.35 – 0.49 | **<0.001** | 0.42 | 0.35 – 0.49 | **<0.001** | 0.42 | 0.36 – 0.49 | **<0.001** | 0.42 | 0.35 – 0.49 | **<0.001** |
| Specialty [Urology] | 0.76 | 0.66 – 0.88 | **<0.001** | 0.76 | 0.66 – 0.88 | **<0.001** | 0.76 | 0.66 – 0.88 | **<0.001** | 0.76 | 0.66 – 0.88 | **<0.001** | 0.76 | 0.66 – 0.88 | **<0.001** |
| Age (years) | 1.09 | 1.08 – 1.11 | **<0.001** | 1.09 | 1.08 – 1.11 | **<0.001** | 1.09 | 1.08 – 1.11 | **<0.001** | 1.09 | 1.08 – 1.11 | **<0.001** | 1.09 | 1.08 – 1.11 | **<0.001** |
| Risk score | 1.00 | 0.99 – 1.02 | 0.585 | 1.00 | 0.99 – 1.02 | 0.583 | 1.00 | 0.99 – 1.02 | 0.579 | 1.00 | 0.99 – 1.02 | 0.716 | 1.00 | 0.99 – 1.02 | 0.710 |
| Prop. Female in practice | 0.90 | 0.88 – 0.92 | **<0.001** | 0.90 | 0.88 – 0.92 | **<0.001** | 0.90 | 0.88 – 0.91 | **<0.001** | 0.90 | 0.88 – 0.91 | **<0.001** | 0.90 | 0.88 – 0.91 | **<0.001** |
| Prop. Black in practice | 1.04 | 1.03 – 1.06 | **<0.001** | 1.04 | 1.03 – 1.06 | **<0.001** | 1.04 | 1.02 – 1.06 | **<0.001** | 1.04 | 1.02 – 1.06 | **<0.001** | 1.04 | 1.02 – 1.06 | **<0.001** |
| Prop. Hispanic in practice | 1.00 | 0.98 – 1.03 | 0.832 | 1.00 | 0.98 – 1.03 | 0.819 | 1.00 | 0.98 – 1.02 | 0.892 | 1.02 | 1.00 – 1.05 | 0.088 | 1.02 | 1.00 – 1.05 | 0.113 |
| Prop. with Medicaid in practice | 1.15 | 1.12 – 1.17 | **<0.001** | 1.15 | 1.12 – 1.17 | **<0.001** | 1.15 | 1.12 – 1.17 | **<0.001** | 1.15 | 1.12 – 1.17 | **<0.001** | 1.15 | 1.12 – 1.17 | **<0.001** |
| Population | 0.96 | 0.94 – 0.98 | **<0.001** | 0.96 | 0.94 – 0.98 | **<0.001** | 0.96 | 0.94 – 0.98 | **<0.001** | 0.97 | 0.95 – 0.99 | **0.003** | 0.97 | 0.95 – 0.99 | **0.003** |
| SDI | 0.99 | 0.97 – 1.01 | 0.279 | 0.99 | 0.97 – 1.01 | 0.275 | 0.98 | 0.96 – 1.00 | 0.082 |  |  |  |  |  |  |
| Metro [Rural] × SDI |  |  |  |  |  |  | 1.11 | 1.03 – 1.20 | **0.005** |  |  |  |  |  |  |
| % Below FPL |  |  |  |  |  |  |  |  |  | 1.01 | 0.96 – 1.06 | 0.678 | 1.02 | 0.97 – 1.07 | 0.453 |
| % Single house |  |  |  |  |  |  |  |  |  | 1.01 | 0.97 – 1.05 | 0.522 | 1.01 | 0.98 – 1.05 | 0.460 |
| % Dropout |  |  |  |  |  |  |  |  |  | 1.02 | 0.98 – 1.07 | 0.288 | 1.03 | 0.98 – 1.08 | 0.201 |
| % No car |  |  |  |  |  |  |  |  |  | 0.97 | 0.93 – 1.00 | 0.053 | 0.96 | 0.93 – 1.00 | **0.037** |
| % Renter occupied |  |  |  |  |  |  |  |  |  | 1.03 | 1.00 – 1.06 | 0.081 | 1.03 | 0.99 – 1.06 | 0.102 |
| % Crowded units |  |  |  |  |  |  |  |  |  | 0.97 | 0.94 – 1.00 | 0.075 | 0.97 | 0.94 – 1.00 | 0.054 |
| % Unemployed |  |  |  |  |  |  |  |  |  | 0.97 | 0.95 – 1.00 | **0.026** | 0.96 | 0.93 – 0.98 | **0.002** |
| % High needs |  |  |  |  |  |  |  |  |  | 0.99 | 0.97 – 1.02 | 0.621 | 0.99 | 0.97 – 1.02 | 0.598 |
| % Black |  |  |  |  |  |  |  |  |  | 1.03 | 1.01 – 1.05 | **0.014** | 1.03 | 1.00 – 1.05 | **0.019** |
| % Hispanic |  |  |  |  |  |  |  |  |  | 0.98 | 0.95 – 1.02 | 0.308 | 0.98 | 0.95 – 1.02 | 0.314 |
| % Foreign born |  |  |  |  |  |  |  |  |  | 1.00 | 0.97 – 1.02 | 0.834 | 1.00 | 0.97 – 1.02 | 0.864 |
| Metro [Rural] × % Unemployed |  |  |  |  |  |  |  |  |  |  |  |  | 1.06 | 1.01 – 1.11 | **0.024** |
| **Random Effects** |  | | | | | | | | | | | | | | |
| σ^2^ | 2.32 | | | 2.32 | | | 2.32 | | | 2.32 | | | 2.32 | | |
| τ_00_ | 0.02 _zip_ | | | 0.02 _zip_ | | | 0.02 _zip_ | | | 0.02 _zip_ | | | 0.02 _zip_ | | |
| τ_11_ |  | | | 0.00 _zip.sdi_ | | | 0.00 _zip.sdi_ | | |  | | |  | | |
| ρ_01_ |  | | | 0.85 _zip_ | | | 0.93 _zip_ | | |  | | |  | | |
| ICC | 0.01 | | | 0.01 | | | 0.01 | | | 0.01 | | | 0.01 | | |
| N | 949 _zip_ | | | 949 _zip_ | | | 949 _zip_ | | | 949 _zip_ | | | 949 _zip_ | | |
| Observations | 11996 | | | 11996 | | | 11996 | | | 11996 | | | 11996 | | |
| Marginal R^2^/ Conditional R^2^ | 0.049 / 0.057 | | | 0.049 / NA | | | 0.049 / NA | | | 0.049 / 0.057 | | | 0.050 / 0.057 | | |

The first model (MLM1) contained SDI, other covariates, and a random intercept. The second model (MLM2) added a random slope for SDI, while the third model (MLM3) included an interaction term between SDI and the metropolitan area. In the fourth model (MLM4) all non-collinear normalized SDOH scores were included rather than the composite SDI in addition to the random intercept. Finally, the fifth model (MLM5) incorporated an interaction between unemployment and the metropolitan area.

MLM: Multilevel model; IRR: Incidence Rate Ratio; CI: Confidence Interval; Metro: Metropolitan area; ID: Infectious Diseases; SDI: Social Deprivation Index; FPL: % Federal Poverty Level; ICC: Intraclass correlation coefficient.
